# Social support mediates the influence of cerebellum functional connectivity strength on postpartum depression and postpartum depression with anxiety

**DOI:** 10.1038/s41398-022-01781-9

**Published:** 2022-02-08

**Authors:** Bochao Cheng, Neil Roberts, Yushan Zhou, Xiuli Wang, Yuanyuan Li, Yiming Chen, Yajun Zhao, Pengcheng Deng, Yajing Meng, Wei Deng, Jiaojian Wang

**Affiliations:** 1 Department of Radiology, West China Second University Hospital of Sichuan University, Chengdu, 610041 China; 2 Huaxi MR Research Center (HMRRC), Department of Radiology, West China Hospital of Sichuan University, Chengdu, 610041 China; 3 Edinburgh Imaging facility, The Queen’s Medical Research Institute (QMRI), School of Clinical Sciences, University of Edinburgh, Edinburgh, EH16 4TJ UK; 4 Department of Nuclear Medicine, West China Hospital of Sichuan University, Chengdu, 610041 China; 5 Key Laboratory of Birth Defects and Related Diseases of Women and Children (Sichuan University), Ministry of Education, Chengdu, 610041 China; 6 Department of Psychiatry, The Fourth People’s Hospital of Chengdu, University of Electronic Science and Technology of China, Chengdu, 610041 China; 7 State Key Laboratory of Primate Biomedical Research, Institute of Primate Translational Medicine, Kunming University of Science and Technology, Kunming, 650500 China; 8 Key Laboratory for NeuroInformation of the Ministry of Education, School of life Science and technology, University of Electronic Science and Technology of China, Chengdu, 625014 China; 9 School of Sociality and Psychology, Southwest Minzu University, Chengdu, 610041 China; 10 Department of Psychiatry, West China Hospital of Sichuan University, Chengdu, 610041 China; 11 Affiliated Mental Health Center & Hangzhou Seventh People’s Hospital, Zhejiang University School of Medicine, Hangzhou, Zhejiang 310063 China

**Keywords:** Depression, Diagnostic markers

## Abstract

Post-Partum Depression (PPD) is the most common health issue impacting emotional well being in women and is often comorbid with anxiety (PPD-A). Previous studies have shown that adequate social support can protect against PPD and PPD-A. However, how the brain connectome is disrupted in PPD and PPD-A and the neural basis underlying the role of social support in PPD and PPD-A remains unclear. The present study aims to explore these issues in patients with PPD and PPD-A. Well-established questionnaires and resting-state functional Magnetic Resonance Imaging (rsfMRI) were performed in 45 PPD, 31 PDD-A patients and 62 Healthy Postnatal Women (HPW). Brain functional integration was measured by analysis of Functional Connectivity Strength (FCS). Association and mediation analyses were performed to investigate relationships between FCS, PPD and PPD-A symptoms and social support. PPD patients showed specifically higher FCS in right parahippocampus, whereas PPD-A patients showed specifically higher FCS in left ventrolateral prefrontal cortex. In all postpartum women, depression symptoms positively correlated with FCS in left paracentral lobule; depression and anxiety symptoms were negatively correlated with FCS in right cerebellem posterior lobe (CPL), a brain region implicated in supporting social cognition and regulation of emotion. Subsequent mediation analysis revealed that perceived social support mediated the association between right CPL FCS and PPD and PPD-A symptoms. Measurement of FCS in disorder-specific neural circuits offers a potential biomarker to study and measure the efficacy of social support for PPD and PPD-A.

## Introduction

Postpartum women experience large changes in physiology, emotional, financial and social support and are susceptible to onset or relapse of mental illnesses such as depression and anxiety [1]. Postpartum depression (PPD) is the most common psychiatric disorder during the postpartum period [2]. In nearly two-thirds of women PPD is comorbid with anxiety disorders (PPD-A) [3]. PPD and PPD-A are a serious public health issue that not only impacts the well being and quality of life of mothers, and family relationships, but also has adverse consequences for their offspring [4, 5]. Effective prevention and timely treatment of PPD and PPD-A could be improved by better understanding of the etiology and the disruptions in neural networks specific to the disorders [6].

The risk factors for PPD and PPD-A are multifactorial. Several psycho-social and biological factors include a history of depression/anxiety, marital difficulties, hormones fluctuations, life stress and inadequate social support etc., are known to be associated with an increased risk of developing PPD [7–9]. Down-regulation of stress responses including dampened sympathetic, hypothalamic-pituitary-adrenal (HPA) axis and inflammatory reactivity to stressors together with appropriate social support have all been proposed as important buffers for stressful life events [10] and a significant relationship has been reported between low levels of perceived social support and severity of PPD and PPD-A [11, 12]. Accumulating evidence indicates that adequate social support could protect against PPD/PPD-A [13, 14]. However, the neural basis underlying the role of social support in PPD and PPD-A remains unclear. Damage to hubs in the brain network which play a key role in the transmission and integration of information [15] is likely to seriously affect network efficiency and integrity [16].

Application of functional Magnetic Resonance Imaging (fMRI) has shown alterations in anterior/posterior cingulate cortex, hippocampus, parahippocampups (PHP), amygdala, insula, striatum and prefrontal brain regions in patients with PPD or PPD-A [17–20]. However, so far there have been no studies of PPD and PPD-A from a whole-brain network perspective. Recent advances in brain connectomics using graph theory have allowed computation of Functional Connectivity Strength (FCS) on a voxel by voxel basis, which is a brain functional integration (FI) measure of the importance of each voxel within whole brain networks [21]. Brain regions with high FCS are considered as functional hubs of the brain for information processing connecting otherwise segregated brain systems [22]. This approach has revealed disruptions in the topological organization of large-scale functional brain networks in patients with depression [23].

In the present study, the data-driven voxel-wise FCS technique was applied to analyze rs-fMRI data collected for first-episode, treatment-naïve patients with PPD and PPD-A, and matched healthy postpartum women (HPW). Patients with PPD and PPD-A were predicted to show abnormal functional integration compared with HPW and of particular interest was to investigate whether social support mediated association between regional FCS and symptoms of postpartum depression and anxiety.

## Materials and methods

### Subjects

The data were collected from a longitudinal project that aimed to investigate the pathogenic factors, prognosis and effective intervention mechanism of patients with postpartum depression (within one year after birth) in Chengdu, China. From 1 June 2018 to 1 January 2020, postpartum women were screened and recruited at the Maternity clinic, West China Second University Hospital of Sichuan University (see Table 1 for details). Forty five drug-naïve patients with PPD, 31 drug-naïve PPD patients with anxiety (PPD-A), and 62 Healthy Postnatal Women (HPW) were recruited at the West China Second University Hospital of Sichuan University, China (Table 1). All participants were right-handed, had full-term, normal puerperium and healthy infants, and matched with respect to age and education level. Diagnosis was made according to the criteria of the Diagnostic and Statistical Manual of Mental Disorders, Fifth Edition (DSM-5) and Chinese Classification and Diagnostic Criteria of Mental Disorders 3rd edition (CCMD-3) by two experienced psychiatrists (YJ and WD) at the Department of Psychiatry, West China Hospital of Sichuan University. Exclusion criteria were presence of cardiovascular disease, diabetes, history of any other Axis I mental disorder such as schizophrenia, bipolar disorder, and substance dependence (other than nicotine), history of suicide, alcohol or drug abuse, history of hormonal contraception, use of psychotropic medications or using vasoactive medications or cognitive behavior therapy and contraindication to MRI. The authors assert that all procedures contributing to this work comply with the ethical standards of the relevant national and institutional committees on human experimentation and with the Helsinki Declaration of 1975, as revised in 2008. All procedures involving human subjects/patients were approved by local ethics committee of West China Second University Hospital of Sichuan University (No. S201731). Written informed consent was obtained from all participants.

**Table 1:** Demographic and clinical characteristics of participants.

	HPW (*n* = 62)	PPD (*n* = 45)	PPD-A (*n* = 31)	*F* value	HPW vs PPD	HPW vs PPD-A	PPD vs PPD-A
Age (years)	32.42 ± 3.92	31.11 ± 3.19	31.03 ± 3.83	*F*_2,135_ = 2.29 (*P* = 0.11)	*P* = 0.069	*P* = 0.11	*P* = 0.92
Education (years)	16.5 ± 1.61	16.69 ± 1.92	16.52 ± 1.57	*F*_2,135_ = 0.18 (*P* = 0.84)	*P* = 0.58	*P* = 0.96	*P* = 0.68
Postpartum time (days)	96.27 ± 58.86	94.29 ± 56.29	100.35 ± 53.16	*F*_2,135_ = 0.11 (*P* = 0.9)	*P* = 0.86	*P* = 0.75	*P* = 0.64
EPDS scores	7.06 ± 4.14	16.2 ± 3.22	18.87 ± 5.46	*F*_2,135_ = 104.07 (*P* < 10^−27^)	*P* < 10^−21^	*P* < 10^−18^	*P* = 0.0092
BAI scores	30.73 ± 7.23	37.18 ± 5.94	53.71 ± 10.23	*F*_2,135_ = 93.83 (*P* < 10^−25^)	*P* < 10^−5^	*P* < 10^−20^	*P* < 10^−12^
PSQ-expected	217.05 ± 29.06	219.87 ± 30.97	211.29 ± 32.18	*F*_2,135_ = 0.74 (*P* = 0.48)	*P* = 0.43	*P* = 0.75	*P* = 0.48
PSQ-perceived	166.06 ± 28.75	147.27 ± 28.64	134.10 ± 28.61	*F*_2,135_ = 14.05 (*P* < 10^−5^)	*P* < 10^−3^	*P* < 10^−5^	*P* = 0.09

One-way analysis of variance (ANOVA) was first used to identify differences in demographics and clinical characteristics. Post-hoc two-sample *t* tests were further used to determine the between group differences in all the indices.

*EPDS* Edinburgh postnatal depression scale, *BAI* Beck’s anxiety inventory, *SSRS* social support rating scale, *PPD* postpartum depression, *PPD-A* postpartum depression with anxiety, *HPW* healthy postnatal women.

### Clinical assessments

Depression, and anxiety, symptoms severity was measured by using the Edinburgh Postnatal Depression Scale (EPDS) [24] and Beck’s Anxiety Inventory (BAI) [25], respectively. In addition, administration of the Postpartum Support Questionnaire (PSQ) [26] allowed measurement of the social support expected and the social support perceived by the postpartum women [27].

### MRI data acquisition

Resting-state fMRI data were acquired on a 3 T MRI system (Skyra, Siemens Healthineers, Erlangen, Germany) at the West China Second University Hospital of Sichuan University. Earplugs were provided to reduce scanner noise and foam padding was used to minimize head motion. Participants were instructed to relax and to remain awake with their eyes closed. Parameters for the Echo Planar Imaging (EPI) sequence were Repetition Time (TR) 3.05 s, Echo Time (TE) 22.5 ms, flip angle = 30°, 36 slices, thickness 4.0 mm, gap 1 mm, voxel size 2.45 × 2.45 × 4.0 mm^3^, matrix size = 94 × 94, and Field of View (FOV) 23 cm × 23 cm.

### fMRI data preprocessing

The rsfMRI data were pre-processed as follows (i) the first 6 volumes were discarded to allow for magnetization equilibrium, (ii) all images were realigned to the first volume to correct head motion and (iii) normalized to the Montreal Neurological Institute (MNI) EPI template and resample to 3 × 3 × 3 mm^3^, (iv) head motion was removed by regression analysis using Friston 24-parameter model, global, white matter and cerebrospinal fluid mean signal intensity and (v) a 0.01–0.1 Hz temporal band-pass filter was applied. No participants showed a maximum displacement of >3 mm or an angular motion of >3^o^ and so none were excluded on account of head motion. Finally, the so-called scrubbing method was applied with linear interpolation to eliminate “bad” images exceeding the pre-set criteria for excessive motion (i.e., Frame Displacement (FD) < 0.5).

### Whole brain voxel-wise functional connectivity strength calculation

Whole brain voxel-wise Functional Connectivity Strength (FCS) was calculated for all gray matter voxels in each participant. FCS is based on graph theory and corresponds to the weighted mean of the degree of centrality over all networks. First, the functional connectivities of each voxel with voxels in the rest of brain were calculated based on the value of Pearson’s correlation coefficient between the relevant time series. Weak connections that may arise from signal noise were removed according to the threshold of <0.25 based on previous studies [28, 29]. Next, the FCS value of a given voxel was computed by averaging all the correlation coefficients higher than the above threshold over the whole brain, and this process was repeated for all voxels to obtain a FCS map for each subject. Finally, prior to statistical and correlation analysis the FCS map was converted to a map of *z* scores and spatially smoothed by using a Gaussian filter with Full Width Half Maximum (FWHM) of 6 mm.

### FCS differences

Analysis of Variance (ANOVA) for FCS was performed to determine whether there were potential differences in brain functional integration among patients with PPD, patients with PPD-A, and HPW. Significance was determined using a cluster-level AlphaSim correction method with *p* < 0.05 (cluster-forming threshold at voxel-level *p* < 0.001). Next, if significant differences in FCS were found, the FCS values in the brain areas showing differences among the three groups were extracted, and post-hoc two-sample *t* tests were applied to identify differences between groups, with significance level set to *p* < 0.05 using Bonferroni correction.

In addition, correlation analysis was performed to investigate the relationships between FCS in those brain regions which showed significant differences between the two groups of patients, and between the patients an controls, and the clinical depression and anxiety scores assessed using the EPDS and BAI inventories and social support assessed using the PSQ questionnaire, and with significance level set to *p* < 0.05.

### FCS-PPD/PPD-A correlation analyses

Whole brain voxel-wise correlation analyses were performed between FCS maps and EPDS, BAI, and other psycho-social factors such as age, education, postpartum time, etc. for all enrolled postpartum women. Significance was determined using a cluster-level AlphaSim correction method with *p* < 0.05 (cluster-forming threshold at voxel-level *p* < 0.001). In addition, mean FCS values were calculated for those brain areas which showed significant correlations with EPDS, and BAI, and ANOVA and post-hoc analyses were performed to determine group differences in FCS of these areas. The significant level was set to *p* < 0.05 with Bonferroni correction.

### Meditation analyses

To investigate whether the relationships between FCS and EPDS, and BAI, were mediated by social support, a meditation analysis was performed using the SPSS macro-PROCESS and bootstrapping approach developed by Hayes [30]. FCS of the brain region(s) specially associated with postpartum depression or anxiety loads was taken as the predictor variable (X), social support was taken as the mediator variable (M), and EPDS or BAI scores were the outcome variables (Y). Path “*a”* represents the relation of X and M, path “*b”* represents the relation of M and Y after controlling for X and path “*c”* represents the relation of X and Y after controlling for M. The variable M is considered to be a mediator if the indirect effect (c – c’ or a × b) is significant. To determine the significance of the mediation, a bootstrapping test (5000 repetitions) was used to generate 95% confidence intervals (CIs), and if the CI did not include the value 0 then the total effect between X and Y was mediated by M. In further analyses, FCS, EPDS and BAI were investigated as potential mediator variables using the same procedure as described above.

## Results

### Demographics and clinical characteristics

There were no significant differences in age (*p* = 0.11), education level (*p* = 0.84), expected social support (*p* = 0.48), or postpartum time (*p* = 0.90) between patients with PPD and PPD-A, or in comparison of patient groups and HPW. Depression and anxiety symptom scores were significantly higher in both PPD and PPD-A patients compared to HPW, and in PDD-A compared to PPD patients. Perceived social support was lower in both PPD and PPD-A relative to HPW, but no significant differences were observed between the PPD and PPD-A patient groups (Table 1).

### FCS differences among patients with PPD and PPD-A, and HPW

**Fig. 1:**
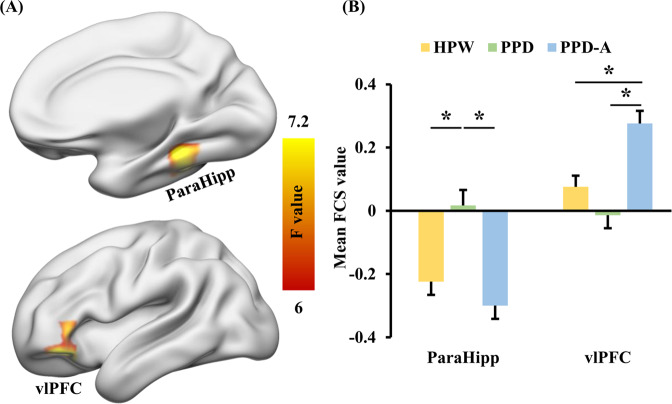
Functional Connectivity Strength (FCS) differences among HPW, PPD, and PPD-A. **A** One-way analysis of variance (ANOVA) of functional connectivity strength (FCS) maps revealed significant differences in right parahippocampus (ParaHipp) and left ventrolateral prefrontal cortex (vlPFC) between postpartum depression (PPD) and PPD with anxiety (PPD-A) and healthy postnatal women (HPW). **B** Post-hoc two-sample *t* tests showed that FCS in ParaHipp was higher in PPD patients compared to PPD-A patients and HPW, and FCS was higher in vlPFC in PPD-A patients compared to both PPD patients and HPW.

Whole brain voxel-wise statistical analyses revealed significant differences in FCS in right parahippocampus (ParaHipp) and left ventrolateral prefrontal cortex (vlPFC) among patients with PPD and PPD-A, and HPW (*p* < 0.001) (Fig. 1A). Post-hoc analyses revealed that the effect in right ParaHipp was due to significantly higher FCS in PPD patients compared to both PPD-A patients and HPW (*p* < 0.05), whereas no significant difference in FCS was found between PPD-A patients and HPW (*p* = 0.24) (Fig. 1B). For the effect in left vlPFC, this was due to significantly higher FCS in PPD-A patients compared to both PPD patients and HPW (*p* < 0.05), whereas no significant difference in FCS was found between PPD patients and HPW (*p* = 0.11) (Fig. 1B). No significant correlations were found between FCS values in ParaHipp and vlPFC and any of the clinical measures.

### Associations between FCS and postpartum depression and anxiety symptom

**Fig. 2:**
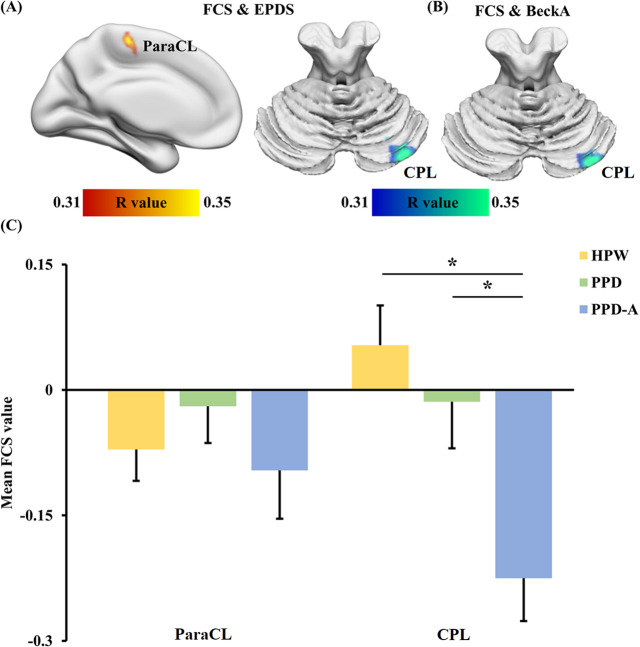
Whole brain voxel-wise correlation analyses were used to identify the associations between FCS and Edinburgh Postnatal Depression Scale (EPDS) and Beck’s Anxiety Inventory (BAI) scores. **A** There is a significant positive correlation between FCS in paracentral lobule (ParaCL), and significant negative correlation between FCS in right cerebellem posterior lobe (CPL), and EPDS, respectively. **B** There is also a significant negative correlation between FCS in right CPL and BAI. **C** Post-hoc two sample t-tests showed that FCS was significantly lower in right CPL in both PPD and PPD-A patients compared to HPW. There was no significant difference in FCS between PPD and PPD-A patients in these or in any other brain regions.

Whole brain voxel-wise correlation analyses in all participants revealed a significant positive relationship between FCS in left paracentral lobule (ParaCL) and EPDS scores (*p* < 0.05, *r* = −0.40) (Fig. 2A) and a significant negative association between FCS in right cerebellem posterior lobe (CPL) and EPDS scores (*p* < 0.05, *r* = −0.37) and BAI scores (*p* < 0.05, *r* = −0.38) (Fig. 2A, B). No other significantly positive correlations were found between FCS maps and other psycho-social factors. Statistical analysis of the mean FCS values in ParaCL and CPL only revealed a significant difference in FCS in right CPL such that FCS in right CPL was lower in both PPD and PPD-A patients groups compared to HPW, but there was no significant difference in FCS between patients with PPD and PPD-A (Fig. 2C).

### Social support mediates the effect of cerebral resting-state FCS on postpartum depression and anxiety

Perceived Social support was negatively correlated with EPDS scores (*r* = −0.44, *p* < 0.001) and BAI scores (*r* = −0.35, *p* < 0.001). FCS values in right CPL were negatively correlated with EPDS scores (*r* = −0.2721, *p* = 0.0012) and BAI score (*r* = −0.258, *p* = 0.0022), and positively correlated with perceived social support measured by PSQ (*r* = 0.2097, *p* = 0.0136). According to the general linear model, FCS in right CPL (*β* = 0.21, *p* < 0.05) and EPDS could be used to predict PSQ scores (*β* = −0.43, *p* < 0.001), and FCS in Cereb (*β* = 0.24, *p* < 0.01) and BAI could be used to predict PSQ scores (*β* = −0.3, *p* < 0.001).

**Fig. 3:**
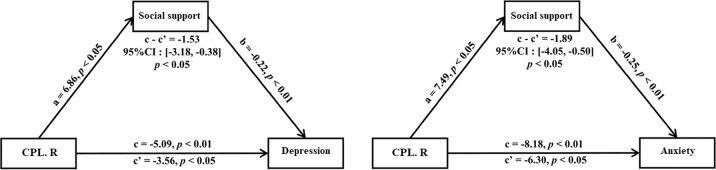
Mediation analysis revealed that FCS of right cerebellem posterior lobe (CPL) affects postpartum depression and anxiety through social support. In particular, social support as measured by the PSQ questionnaire mediates the effect of right CPL on Edinburgh Postnatal Depression Scale (EPDS) and Beck’s Anxiety Inventory (BAI) scores.

Meditation analyses further revealed that social support plays a meditating role in the association between FCS in right CPL and both EPDS and BAI score in all postpartum women (EPDS: 95% CI = (−3.18, −0.38), *p* < 0.05; BAI: 95% CI = (−4.05, −0.50), *p* < 0.05; Fig. 3). In addition, we also exchanged other measures as mediator, but no significant meditation effects were found.

## Discussion

Three main findings were obtained from the measurement of regional functional integration (FI) by FCS in rsfMRI data acquired for patients with PPD and PPD-A, and HPW, and subsequent correlation and mediation analyses. First, PPD group showed specifically higher FCS in right ParaHipp, and PPD-A group showed specifically higher FCS in left vlPFC, compared with the respective other patient group and HPW. Second, all enrolled postpartum women had a significant positive correlation between FCS in left ParaCL and postpartum depression loads, and a significant negative correlation between FCS in right cerebellem posterior lobe (CPL) and postpartum depression loads and postpartum anxiety loads. Third, perceived social support mediated the influence of FCS in right CPL on depression and anxiety symptom. Taken together these findings provide evidence of abnormal brain functional interactions in patients with PPD and PPD-A and the importance influence of lack of social support in these conditions. By determining the underlying mechanism for these brain alterations it may be possible to develop more effective therapies and treatment strategies.

The findings of the present study show that PPD and PPD-A patients have disorder specific alterations in the FI of the brain network. This is supported by reports that ParaHipp was found with higher activation in female depressive patients than healthy controls [31], and is involved in processing negative emotional stimuli, including those related to trauma [32]. Furthermore, lesions in temporal cortex and ParaHipp, which are part of the temporal-limbic network, typically disrupt episodic memory which is common in patients with depression and cognitive impairment [33]. On the other hand, increased functional connectivities of ParaHipp with other structures such as subgenual cingulate gyrus and dorsolateral prefrontal cortex have been reported in patients with depression [34]. This suggests that deficits in the ventromedial neural system may disrupt early stimulus appraisal, encoding, and regulation of emotion [35]. FCS abnormalities in ParaHipp may impair emotional regulation and disrupt memory function in patients with depression [36].

Furthermore, FCS was significantly increased in left vlPFC in patients with PPD-A compared to patients with PPD and HPW. Hearing an infant crying has been linked to heightened anxious responses and vlPFC hyperactivity in early postpartum mothers [37]. Specifically, vlPFC modulates amygdala responses to threats in order to maintain goal-directed behavior [38, 39]. On the contrary, in a task fMRI study of viewing negative pictures, vlPFC hypoactivity was associated with reduction of subjective distress in anxiety-prone participants [40]. Therefore, vlPFC hyperactivity in PPD-A patients may be a compensatory mechanism to support processing of negative emotions and regulation of anxiety symptoms [41, 42], especially when mothers confront challenges and threats in the postpartum period, such as insufficient social support.

In the current study, a negative correlation was observed between FCS in right CPL and postpartum depression and anxiety scores in all postpartum women. There is substantial evidence to indicate a crucial role for the cerebellum in social-cognition and affective functions through its relationship with prefrontal cortices and other brain structures (e.g., parietal lobe and temporal lobe) and which suggest that the cerebellum (especially the CPL) acts as a relay station in the limbic system that is important for regulating emotion [43]. Compared to healthy controls, patients with depression have been reported to have reduced gray matter volume [44, 45], and increased FCS [29] in the CPL. Lesions of the CPL were reported to reduce pleasure in response to positive stimuli [46], and which may produce emotional passivity, personality changes and psychotic symptoms [47]. In addition, CPL lesions result in the cerebellar cognitive affective syndrome, characterized by dysfunction in execution, linguistic skills and affective regulation of affect etc.

Importantly, we found that perceived social support mediated the influence of FCS in right CPL on depression and anxiety symptoms in all postnatal women. Behaviorally, the association of social support with depression and anxiety symptoms has been well established in previous studies [13, 48–50], and which is consistent with the findings of significant correlations between social support and depression (*r* = −0.44, *p* < 0.001) and anxiety (*r* = −0.35, *p* < 0.001) scores in the present study. The cerebellum, particularly the CPL, involves in intellect and emotion control function, and is an important hub in the social cognition network through functionally connected with mid-brain and neocortical areas [51], and supports brain-wide maturation of flexible and social behaviors (i.e., social cognition). Adequate and subtle social interaction requires one to be able to infer the emotion of another person, and which is mediated by the cerebellum [52]. All these findings demonstrated that, at the connectome level, variance in postpartum symptoms of depression and anxiety could potentially be related to abnormal in functional connectivity of right CPL. Furthermore, social support serves as a potential mechanism to explain the impact of FCS in right CPL on postpartum depression or anxiety.

The present study has several limitations that should be acknowledged. First, the influence of several potentially important factors were not controlled for or studied, including natural or assisted conception, natural delivery versus cesarean section, hormones fluctuations, cognitive functions and whether patients were primipara or multipara. These factors and sub-group analysis should be included in future studies. Second, the PPD and PPD-A groups are likely to include patients representing a number of phenotypes in terms of timing, severity and the exact nature of symptoms. Study of the effect of post-partum time may be an important topic for identification of associations between clinical symptoms and postpartum time. Third, the analysis was based only on measurement of FCS in resting-state fMRI data and in future studies it will be interesting to incorporate multimodal neuroimaging data. Finally, because of the cross-sectional design it is not possible to draw conclusions regarding a potential causal relationship between social support, fMRI functional connectivity, PPD and PPD-A. Longitudinal studies with multimodal MRI data including other measures of brain function (e.g., task-related fMRI) and structure (e.g., gray matter volume and surface area) are warranted to better reveal the neuropathology of the PPD and PPD-A phenotypes and determine if there are causal relationships between brain changes, symptoms and social support.

In conclusion, specific alterations of FCS were observed in ParaHipp and vlPFC in PPD and PPD-A patients relative to HPW. Furthermore, alterations in FCS in ParaCL and CPL were found to be associated with postpartum depression and anxiety symptom. The present study also provides initial evidence that perceived social support mediates the relationship between FCS of the CPL and postpartum depression and anxiety loads. Taken together these findings suggest that social support is important in maintaining mental health of postpartum women. In future work it may be possible to develop imaging methods for predicting how perceived social support influences postpartum depression and anxiety symptoms in individual patients.
